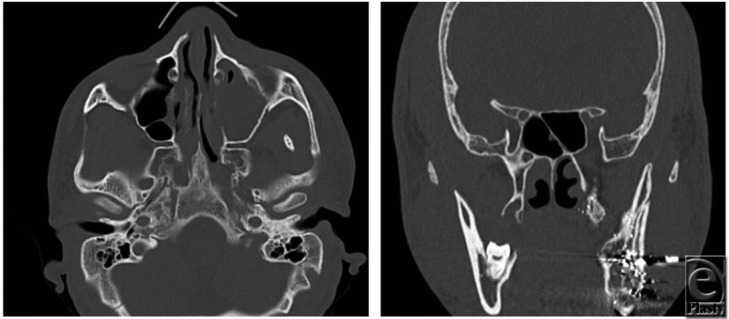# Zygomatic Arch Fracture With Coronoid Impingement

**Published:** 2015-01-22

**Authors:** Gerhard S. Mundinger, Michael Daniel, Justin M. Sacks

**Affiliations:** Department of Plastic and Reconstructive Surgery, Johns Hopkins Hospital, Baltimore, MD

**Keywords:** zygoma, zygomatic arch fracture, coronoid impingement, trismus, facial trauma

**Figure:**
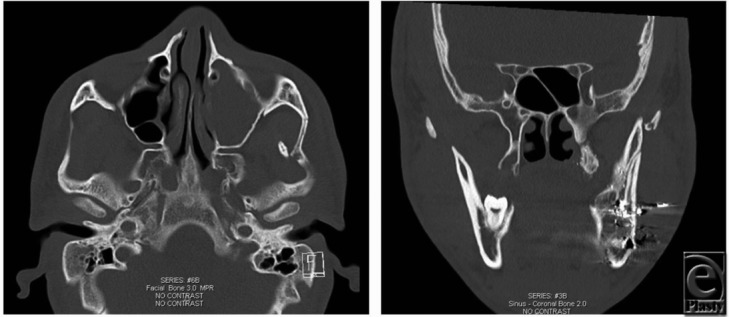


## DESCRIPTION

A 20-year-old man with a previous gunshot to the left mandible presented with left facial swelling and trismus after being assaulted in the face with a closed fist. Mouth opening from maxillary to mandibular central incisors was limited to 3 cm. Dedicated maxillomandibular computed tomographic (CT) scan demonstrated an isolated left zygomatic arch fracture impinging the coronoid of the left mandible.

## QUESTIONS

1. **What is the incidence and mechanism of zygomatic arch fractures?**
2. **What are the indications for isolated arch fracture reduction?**
3. **What is the mechanism of coronoid impingement by zygomatic arch fractures?**
4. **What are the common surgical approaches to reduce isolated zygomatic arch fractures?**

## DISCUSSION

Zygomatic arch fractures are common injuries following blunt trauma, being present in 11% to 15% of patients with other concomitant facial fractures in large series.^1^ Isolated unilateral zygomatic arch fractures are less common, accounting for 3% to 5% of isolated blunt mechanism facial fractures.^1^ Isolated unilateral arch fractures are typically caused by low energy anteroposterior or lateral impacts and are not associated with polysystem trauma. In contrast, isolated bilateral arch fractures are extremely rare, are typically caused by high-energy posterior impacts, and carry a grave prognosis due severe associated injuries.^2^ All patients presenting with facial trauma should have craniofacial CT imaging to evaluate facial fractures and associated injuries.

Typically, displaced isolated fractures of the zygomatic arch result in flattening of the cheek, noticeable lateral midface depression, and asymmetric reduction of facial width. Arch projection is a crucial component of facial esthetics, as it dictates facial width and is a key component of cheek projection. Displaced arch fractures should be reduced to improve facial esthetics, but repair is not urgent in the absence of trismus or coronoid impingement. Reduction should occur in the first 1 to 2 weeks postinjury in adults, and within the first week in children^3^ to avoid malunion and subsequent need for corrective osteotomies. Closed reduction is generally not advisable for displaced isolated arch fracture treatment, and arch plating is not routinely necessary,^4^ as an adequately reduced and positioned arch rests in a stable fascial and muscular sling without displacing forces.^5^

Coronoid impingement can be best understood by considering key regional anatomy. The zygomatic arch is formed by the temporal process of the zygoma and zygomatic process of the temporal bone, which meet at the zygomaticotemporal suture at the midpoint of the arch. In addition, 2 key muscles of mastication, the temporalis, which runs deep to the arch from its superior origin in the temporal fossa inserting on the mandibular coronoid process, and the masseter, which originates from the arch, are functional elements intimately linked to arch position. In patients with zygomatic arch fractures and limited mouth opening (interincisal distance <40 mm), the arch may be in direct bone-on-bone contact with the coronoid process of the mandible, and urgent open reduction is indicated. Impingement is suspected clinically in the presence of trismus with limited mouth opening and is confirmed radiographically with CT imaging.

Open reduction is the mainstay of isolated arch fracture treatment. Two common techniques include the Gillies and Keen approaches.^4^ In the Gillies approach, an incision is made within the temporal hairline and carried just deep to the deep temporal fascia.^6^^-^7 An elevator is then positioned immediately deep to the deep temporal fascia and slid inferiorly into position deep to the zygomatic arch at the fracture site. In the Keen approach, an intraoral incision is made in the upper gingival buccal sulcus, and the fracture is accessed directly through posterosuperior dissection in a subperiosteal plane along the zygomaticomaxillary buttress to a point deep to the zygomatic arch and fracture site.^8^ In both approaches, the nondominant hand is utilized to direct the elevator to the fracture site deep to the arch and aids the dominant hand in controlled fracture reduction. Intraoperative or postoperative imaging is recommended to confirm adequacy of open reduction.

In summary, isolated zygomatic arch fractures should generally be managed with open reduction without plating within 2 weeks of injury to restore facial esthetics. In the setting of traumatic coronoid impingement (trismus with limited mouth opening, and bone-on-bone contact between the coronoid and displaced arch fracture), arch reduction should be performed urgently. In the case of coronoid impingement presented here, the patient's arch fracture was reduced through a Keen approach on the day of presentation with improved postoperative mouth opening to 6 cm.

**Figure:**